# Local Attitudes towards Postgraduate Psychiatry Training: A Maltese Perspective

**DOI:** 10.1192/j.eurpsy.2022.2194

**Published:** 2022-09-01

**Authors:** E. Camilleri, B. Grech

**Affiliations:** Mount Carmel Hospital, Psychiatry, Attard, Malta

**Keywords:** PostgraduateTraining, Improvements, Ideas, Malta

## Abstract

**Introduction:**

The updated programme for postgraduate psychiatry training in Malta was implemented in 2017. The trainees’ perspective was identified as an important and untapped resource in implementing positive change.

**Objectives:**

1. Identification of lacunae within the training programme 2. Acquire ideas regarding new initiatives 3. Identify what is particularly good. 4. Present a comprehensive report to the relevant stakeholders 5. Use of findings to work on concrete changes, with re-audit in 1 year.

**Methods:**

Data from a national online survey of 12 Maltese Psychiatric Trainees from a total of 19 (63%% response rate) were examined. Both qualitative and quantitative data was gathered by making use of Likert scales as well as open ended questions. Various areas were surveyed, including 1. Ease of accessibility and quality of Clinical Supervision & Educational Supervision 2. Lectures and Teaching Seminars 3. After hours Duties 4. Psychotherapy Module 5. Preparation for MRCpsych Examinations

**Results:**

A noticeable difficulty in accessing clinical supervision (66%), the lack of research presentation opportunities (75%), as well as difficulties in the psychotherapy module (66.7%) were noted.

**Conclusions:**

All results were collated into a six-page report. This report was presented to both the Maltese Postgraduate Training Committee as well as the Executive committee of the Maltese Association of Psychiatry and the Chairman of the Psychiatry Department. Various suggestions were flagged for Implementation including: 1. Rotation specific teaching 2. Restructure of the Psychotherapy module including training 3. Annual Research Day 4. Complex Case Discussions Follow up plans include reaudit in one year following the implemented changes.

**Disclosure:**

No significant relationships.

